# Supplementary material for: Transcriptome Analysis Reveals Vimentin-Induced Disruption of Cell–Cell Associations Augments Breast Cancer Cell Migration
Source: Cells. 2022 Dec 13;11(24):4035. doi: 10.3390/cells11244035 (PMC9776984; doi:10.3390/cells11244035)
Supplement: Supplementary file 1 [file cells-11-04035-s001.zip › DEGs and enrichment analysis.zip!/DEGs and enrichment analysis/WT_VIMvsVector KEGG/UP/WT_VIMvsVector_UP.pathway_map.zip!/WT_VIMvsVector_UP.pathway_map/src/hsa04110.html]

hsa04110


- hsa:4176

- Up regulated genes

ENSG00000166508(2.12068744963158)
- hsa:4171

- Up regulated genes

ENSG00000073111(2.4296698586728)
- hsa:4174

- Up regulated genes

ENSG00000100297(2.27874078513338)

- hsa:4998

- Up regulated genes

ENSG00000085840(2.11209478685696)

- hsa:4998

- Up regulated genes

ENSG00000085840(2.11209478685696)

- hsa:4176

- Up regulated genes

ENSG00000166508(2.12068744963158)

- hsa:4174

- Up regulated genes

ENSG00000100297(2.27874078513338)

- hsa:4171

- Up regulated genes

ENSG00000073111(2.4296698586728)

- hsa:5347

- Up regulated genes

ENSG00000166851(2.38554234415561)

- hsa:10459

- Up regulated genes

ENSG00000116670(1.93023390989801)

- hsa:9088

- Up regulated genes

ENSG00000127564(2.77874383365084)

- hsa:9700

- Up regulated genes

ENSG00000135476(1.9754235673444)

- hsa:991

- Up regulated genes

ENSG00000117399(2.41242866521263)

- hsa:8318

- Up regulated genes

ENSG00000093009(2.56188132877989)

- hsa:993

- Up regulated genes

ENSG00000164045(1.78085789611929)

- hsa:9133

- Up regulated genes

ENSG00000157456(1.60409075038332)

- hsa:890

- Up regulated genes

ENSG00000145386(1.72013468925064)

- hsa:890

- Up regulated genes

ENSG00000145386(1.72013468925064)

- hsa:1870

- Up regulated genes

novel.507(3.55620908288126)
- hsa:1869

- Up regulated genes

ENSG00000101412(1.977544660594)

Close
